# Factors related to the prevalence of pregnancy complications among subsistence workers in Medellín - Colombia

**DOI:** 10.47626/1679-4435-2023-1143

**Published:** 2025-01-31

**Authors:** María Osley Garzón Duque, Paula Andrea Uribe Cardenas, Fabio León Rodríguez-Ospina, Manuela Jiménez Cifuentes, Valentina Zapata Paz

**Affiliations:** 1 Faculty of Medicine, Universidad CES, Medellín, Antioquia, Colombia; 2 National Faculty of Public Health, Universidad CES, Medellín, Antioquia, Colombia

**Keywords:** mujeres trabajadoras, sector informal, vulnerabilidad social, salud pública, complicaciones del embarazo, working women, informal sector, social vulnerability, public health, pregnancy complications

## Abstract

**Introduction:**

For the informal female workers on the streets and sidewalks of big cities, called “venteras,” experiencing a pregnancy in a healthy environment is an ideal difficult to achieve.

**Objectives:**

To determine the prevalence of pregnancy complications and their relationship with health, working, and non-working conditions among the “venteras” in downtown Medellín.

**Methods:**

This cross-sectional study utilized primary data collected through a survey administered to 291 workers. An assisted survey was conducted after obtaining informed consent from the participants. The variables included self-reported pregnancy complications, sociodemographic data, work-related conditions, home responsibilities, environmental factors, support and solidarity, and comorbidities. Descriptive and bivariate analyses were performed using chi-square tests and prevalence ratios (PR). A multivariate analysis was conducted using binomial regression. Statistical tests were carried out with 95% confidence intervals (95% CI) and a 5% margin of error.

**Results:**

The workers’ mean age was 48 years, with a mean education of < 6 years; 39% had more than 2 dependents. Additionally, 53.6% reported feeling discriminated against by authorities, and 23.7% by their peers; 21.6% reported severe family dysfunction, and 15.5% showed moderate-to-severe depressive symptoms. The prevalence of complications during pregnancy was 36.9%. Factors contributing to the explanation (p < 0.05) of more complications included living in a room/boarding house (_Adjusted_PR [_A_PR] = 3.78, 95% CI 1.20-11.91), working while pregnant (_A_PR 1.80, 95% CI 1.07-3.03), having moderate/severe depressive symptoms (_A_PR = 15.02, 95% CI 1.34-167.79), and exposure to pollutants for more than 10 hours a day (_A_PR = 8.11, 95% CI 8.04-214.09).

**Conclusions:**

Conditions associated with a higher prevalence of pregnancy complications in these workers are health determinants that require joint efforts from the state, workers, and society.

## Abstract (es)

**Introducción:**

Para las trabajadoras informales “venteras” en las calles y aceras de las ciudades, vivir un proceso de gestación en un ambiente saludable es un ideal difícil de alcanzar.

**Objetivos:**

Determinar la prevalencia de complicaciones en el embarazo y su relación con las condiciones de salud, laborales y extralaborales en las trabajadoras informales “venteras” del centro de Medellín.

**Métodos:**

Estudio transversal con fuente primaria de información tomada por censo a 291 trabajadoras. Se aplicó encuesta asistida, previa toma de consentimiento. Las complicaciones autorreportadas en el embarazo se relacionaron con variables de condiciones sociodemográficas, del trabajo, responsabilidades en el hogar, ambientales, de apoyo y solidaridad, y comorbilidades. Se realizaron análisis descriptivo y bivariados con prueba chi cuadrado y razones de prevalencia (RP), además de análisis multivariado con regresión binomial y pruebas estadísticas con intervalos de confianza del 95% (IC95%) y error del 5%.

**Resultados:**

Las trabajadoras tenían una edad promedio de 48 años y menos de 6 años de escolaridad; el 39% tenía más de dos personas a su cargo, el 53,6% percibía discriminación por parte de las autoridades y el 23,7% de sus compañeros; el 21,6% presentaba disfuncionalidad familiar severa y el 15,5% sintomatología depresiva moderada-severa. La prevalencia de complicaciones en embarazo fue del 36,9%. Aportaron a la explicación (p < 0,05) de mayores complicaciones: vivir en cuarto/inquilinato (RP_Ajustada_ [RP_A_] = 3,78, IC95% = 1,20-11,91), trabajar embarazadas (RP_A_ = 1,80, IC95% = 1,07-3,03), tener sintomatología depresiva moderada/grave (RP_A_ = 15,02, IC95% = 1,34-167,79) y exponerse más de 10 horas a un foco contaminante (RP_A_ = 8,11, IC95% = 8,04-214,09).

**Conclusiones:**

Las condiciones que se asocian y explican mayores prevalencias de complicaciones en el embarazo de estas trabajadoras son determinantes de la salud que requieren acciones combinadas del Estado, los trabajadores y la sociedad.

## INTRODUCTION

Pregnancy is a crucial period for women and their families, in which maintaining the physical and mental health of the mother-to-be can contribute to a successful birth. Although women require a stress-free environment, achieving this can be challenging for working women since the number of employed women is increasing globally, and approximately 90% of them continue working during pregnancy.^1,2^ This is due, in part, to social and economic changes in the urbanization process, which create more job opportunities.^3^ Engaging in paid work outside the home during pregnancy may have an impact on obstetric and perinatal outcomes. Previous studies^1-5^ have shown that factors such as long working hours, prolonged standing, lifting significant weights, or heavy workloads^6^ may increase the risk of obstetric complications.

These factors are also present among subsistence workers. However, evidence remains scarce on both the prevalence of pregnancy complications and the associated work and non-work factors. In Latin America and the Caribbean, employment inequalities continue to represent a large gap between men and women.^7^ Although the indicators presented by the International Labour Organization (ILO) in 2017 and 2018 reflect improvements in some employment and occupational conditions, informality remains more common among women, agricultural workers, and people with lower education.^7^

People who work informally on the streets and sidewalks of cities in subsistence jobs^8^ are typically at the extremes of life^7^: young people aged 14 to 24 years and adults over 64 years of age (representing 78%), including many women of childbearing age,^5^ who may become pregnant and be forced to continue working for subsistence. However, there is insufficient evidence to describe or indicate the prevalence of pregnancy complications or how they relate to work and non-work factors.

Pregnancy complications include hypertensive disorders,^3,6,9^ threat of preterm labor or miscarriage,^1,4,6^ complete abortion,^2,3,10^ delivery-related complications,^3,6^ small for gestational age or low birth weight (LBW),^2,3^ and prenatal maternal depression.^11^ These complications are related to workload, occupational, environmental, and psychological exposures, as well as to various sociodemographic factors that can be decisive in the development of pregnancy complications. However, there is little evidence on these aspects in informal workers, such as the “venteras” on the streets of downtown Medellín, Colombia.

A cross-sectional study conducted in 1999 in Mexico City, which included 426 pregnant street vendors, showed a higher risk of having a LBW infant among those having a daily commute of more than one and a half hour. This risk was also increased in women who had no control over the amount of products to be sold, those selling second-hand products without proper infrastructure to display them, those who carried or placed the products on the ground, and those who asked for financial support to start their business.^5^

For the reasons outlined above, this study aimed to determine the prevalence of self-reported pregnancy complications among women engaged in subsistence work (informal workers called “venteras”) on the streets of Medellín and to examine their relationship with health and working conditions, support and solidarity from colleagues and the state, and non-working conditions in the year 2016.

## METHODS

A cross-sectional study was conducted using primary data obtained through a survey of 291 “venteras” in downtown Medellín performed for the doctoral dissertation titled: “Vulnerabilidad socio laboral y ambiental de un grupo de trabajadores informales “venteros” del centro de Medellín, bajo el modelo de fuerzas motrices. Medellín 2015-2019.”

The study included female workers over 18 years of age, with more than 5 years working as a “ventera,” who were informed of the study and its procedures and provided their informed consent prior to data collection. No workers were excluded according to the defined criteria.

### VARIABLES

Self-reporting of pregnancy complications was considered a dependent variable and was assessed with the following question: “Have you experienced or are you currently experiencing any complications with your pregnancy?”, categorized as: 1. Yes; 2. No.

### INDEPENDENT VARIABLES

Sociodemographic characteristics, working conditions, workplace support, household responsibilities, chronic degenerative diseases, diseases related to air and noise pollution, and depressive symptoms, screened using the Zung Scale validated for use in adults in Colombia,^12^ were included. This scale allows for the identification of individuals at risk for depression, with cutoff points defined by Conde et al.^12^ as follows: 10-20 indicate no depression; 36-51 indicate subclinical depression and normal variants; 52-67 indicate moderate-to-severe depression; and 68-80 indicate severe depression. Age, sex, and seniority as a “ventera” were considered confounders and controlled for in bivariate and multivariate analyses.

### DATA COLLECTION PROCESS

The survey was administered by the principal investigator and a public health professional, who served as a fieldwork assistant, after conducting a pilot test for standardization. Selection bias was controlled by surveying all women participating in the study, and information bias was controlled by standardizing data collection using an instrument that was developed, validated, and standardized in terms of form and content with the workers, and later reviewed by experts in the field. All participants were surveyed at their sales points, in union meetings, and in other social interaction spaces.

### DATA ANALYSIS

An exploratory analysis was conducted considering the nature and level of measurement of the variables. In bivariate analysis, the chi-square test of association and prevalence ratio (PR) were used to assess the strength of association between the prevalence of self-reported pregnancy complications and other characteristics and conditions under study, with their respective 95% confidence interval (95% CI). In multivariate analysis, a binary logistic regression was used for explanatory purposes, adjusting for confounders to identify the factors that could better explain pregnancy complications. All characteristics and conditions that were significantly associated with pregnancy complications or presented p-values < 0.25 in the bivariate analyses were included, according to the Hosmer-Lemeshow test. All tests were performed with 95% CI and a 5% margin of error. The calculations were performed using SPSS 21, licensed by the Universidad CES, Epidat 3.1, and Excel.

The study was classified as minimal risk. This article is a byproduct of the doctoral dissertation mentioned in the first paragraph of the Methods, approved by the Institutional Research Ethics Committee through act No. 84 dated September 24, 2015. The study adhered to ethical principles (Resolution 008430 of 1994 - Ethics for research involving human participants in Colombia) and the 1975 Declaration of Helsinki.

## RESULTS

### SOCIODEMOGRAPHIC AND ECONOMIC STATUS OF THE FEMALE WORKERS

The workers’ mean age was 48.35 (SD 12.05) years. Overall, 50% had 5.7 years or less of schooling and a monthly income of $500,000 (Rq: $400,000) or less. In addition, 84.5% (246 participants) were mothers and the head of the household, 57% (166) lived in a house, 49.1% (143) paid rent, and 14.1% (41) shared their homes with other families. Furthermore, 51.9% (151) considered that their home was in poor or fair condition, and 75.6% (220) lived in homes within the low and very low socioeconomic strata (Table 1).

**Table 1:** Pregnancy complications associated with sociodemographic data and household responsibilities among informal workers called “venteras” in downtown Medellín, 2016

Sociodemographic data	Pregnancy complications		Total	Chi-square (p-value)	PR (95% CI)
	Yes (n/%)	No (n/%)			
Recategorized age (4 groups) (years)					
18 to 29	10 (55.6)	8 (44.4)	18	4.49 (0.210)	1.0
30 to 44	34 (36.6)	59 (63.4)	93		0.66 (0.40-1.08)
45 to 59	44 (38.3)	71 (61.7)	115		0.69 (0.43-1.11)
60 or more	13 (27.7)	34 (72.3)	47		0.50 (0.27-0.90)
Marital status					
With a partner	45 (39.5)	69 (60.5)	114	0.51 (0.472)	1.12 (0.52-1.83)
Without a partner	56 (35.2)	103 (64.8)	159		1.0
Average monthly income (COP)					
≤ 500,000	58 (39.7)	88 (60.3)	146	1.00 (0.317)	1.17 (0.86-1.61)
> 500,000	43 (33.9)	84 (66.1)	127		1.0
Dependents					
> 2	49 (41.9)	68 (58.1)	127	2.10 (0.150)	1.26 (0.92-1.70)
≤ 2	52 (33.3)	104 (66.7)	117		1.0
Person who contributes the most to the household					
Yes	88 (37.1)	149 (62.9)	237	0.01 (0.906)	1.03 (0.64-1.64)
No	13 (36.1)	23 (63.9)	36		1.0
Type of family					
Nuclear	27 (45.0)	33 (57.0)	60	7.92 (0.160)	1.0
Extended	18 (47.9)	23 (56.1)	41		0.97 (0.62-1.52)
Reconstituted	24 (40.7)	35 (59.3)	59		0.90 (0.59-1.37)
Lone parent	7 (22.7)	24 (77.4)	31		0.50 (0.25-1.01)
Single parent	24 (32.0)	51 (68.0)	75		0.71 (0.46-1.09)
Blended	1 (14.3)	6 (85.7)	7		0.32 (0.05-1.99)
Type of housing					
House	53 (34.0)	103 (66.0)	156	7.44 (0.114)	1.0
Apartment	34 (37.4)	57 (62.6)	91		1.10 (0.78-1.55)
Room	4 (36.4)	7 (63.6)	11		1.07 (0.48-2.41)
Boarding house	5 (55.6)	4 (44.4)	9		1.64 (0.88-3.05)
Other	5 (83.3)	1 (16.7)	6		2.45 (1.61-3.73)
Lives with other families					
Yes	16 (41.0)	23 (59.0)	39	0.33 (0.563)	1.13 (0.75-1.71)
No	84 (36.2)	148 (63.8)	232		1.0
Responsible for children or older people					
Yes	82 (39.4)	126 (60.6)	208	3.83 (0.050)	1.10 (1.01-1.20)
No	6 (20.7)	23 (79.3)	29		1.0
In charge of people who require special care					
Yes	20 (50.0)	20 (50.0)	40	2.05 (0.151)	1.33 (0.92-1.91)
No	64 (37.6)	62 (4.0)	170		1.0
Feels unconcerned about leaving children at home unsupervised					
Always	45 (38.5)	72 (61.5)	117	3.17 (0.365)	1.0
Almost always	4 (66.7)	2 (33.3)	6		1.73 (0.94-3.19)
Rarely	19 (48.7)	20 (51.3)	39		1.27 (0.85-1.88)
Never	8 (50.0)	8 (50.0)	16		1.30 (0.76-2.23)
Has been unable to work due to household issues					
Yes	35 (45.5)	42 (54.5)	77	0.39 (0.530)	1.11 (0.80-1.56)
No	42 (40.8)	61 (59.2)	103		1.0
Daily hours spent on housework					
More than 4	20 (39.2)	31 (60.8)	51	0.13 (0.72)	1.07 (0.73-1.58)
4 or less	81 (36.5)	141 (63.5)	222		1.0

COP = Colombian peso; PR = prevalence ratio.

### WORKING CONDITIONS

Half of the participants worked 10 (Rq 3) hours a day, at least 6 days a week, and 50% of them had been working for 20 years or more. Among all participants, 93.8% (273) reported that being a “ventera” was their only source of income. However, 43% (125) did not have permission to work in public spaces. Approximately 29% of them had been housekeepers before becoming street vendors, and for 23% (67), this had been their only occupation (Table 2).

**Table 2:** Pregnancy complications associated with occupational, environmental, and support or solidarity exposures of informal workers called “venteras” in downtown Medellín, 2016

Work-related factors	Pregnancy complications		Total	Chi-square (p-value)	PR (95% CI)
	Yes (n/%)	No (n/%)			
Worked during pregnancy					
Yes	65 (43.6)	84 (56.4)	141	6.18 (0.013)	1.50 (1.08-2.09)
No	36 (29.0)	88 (71.0)	124		1.0
Number of working days in a week					
6-7	96 (36.2)	169 (63.8)	265	1.31 (0.250)	0.57 (0.33-1.01)
5 or less	5 (62.5)	3 (37.5)	8		1.0
Seniority as a “ventera” (years)					
More than 20	45 (40.9)	65 (59.1)	110	1.21 (0.270)	1.19 (0.87-1.62)
20 or less	56 (34.4)	107 (65.6)	163		1.0
Feels she has to work harder than her male peers					
Yes	57 (41.0)	82 (59.0)	139	1.71 (0.190)	1.23 (0.90-1.68)
No	44 (33.3)	88 (66.7)	132		1.0
Perception of respect from peers					
No	16 (47.1)	18 (52.9)	34	1.55 (0.210)	1.30 (0.88-1.94)
Yes	85 (36.0)	151 (64.0)	236		1.0
Perception of peer support					
No	24 (36.4)	42 (63.6)	66	0.02 (0.896)	0.97 (0.68-1.40)
Yes	76 (37.3)	128 (62.7)	204		1.0
Perception of discrimination by peers					
Yes	33 (47.8)	36 (52.2)	69	4.41 (0.036)	1.42 (1.04-1.94)
No	68 (33.7)	134 (66.3)	202		1.0
Perception of discrimination by the state					
Yes	64 (41.0)	92 (59.8)	156	2.21 (0.136)	1.28 (0.92-1.77)
No	37 (32.7)	78 (67.8)	115		1.0

PR = prevalence ratio.

A total of 86.8% (249) perceived that their colleagues treated them with respect and consideration. However, 26% (75) reported feeling discriminated against, primarily by authorities (60%). In addition, 58.7% (169) had parents or relatives who had been or were street vendors (Table 2).

### HOUSEHOLD RESPONSIBILITIES

Half of the participants had at least 2 dependents, 62.2% (117) had children attending school, and more than half of them helped children with their schoolwork. Half of the participants had someone to help with household chores and dedicated 2.10 or more hours per day to these tasks. Out of every 100 workers, 50 had been balancing household chores with street vending for 15 years. A total of 62.2% (115) had to bring the children to their sales point, and 43% had, at some point, missed work due to the lack of someone to take care of their children (Table 2).

### FAMILY DYSFUNCTION, EMOTIONAL SYMPTOMS, AND PREVALENCE OF PREGNANCY COMPLICATIONS

A total of 21.6% (63) of the “venteras” reported severe family dysfunction, while 19.9% (58) reported having mild-to-moderate family dysfunction. Additionally, 5.8% (17) had moderate-to-severe anxiety symptoms, and 15.5% (45) had moderate-to-severe depressive symptoms (Table 3).

**Table 3:** Pregnancy complications associated with health conditions in informal workers called “venteras” in downtown Medellín, 2016

Health conditions	Pregnancy complications		Total	Chi-square (p-value)	PR (95% CI)
	Yes (n/%)	No (n/%)			
Body mass index					
Overweight/obesity	68 (34.2)	131 (65.8)	199	2.51 (0.112)	0.77 (0.56-1.05)
Low/normal weight	33 (44.6)	41 (55.4)	74		1.0
Diabetes					
Yes	4 (21.1)	15 (78.9)	19	1.50 (0.221)	0.54 (0.22-1.34)
No	43 (38.7)	68 (61.3)	111		1.0
Hypertension					
Yes	20 (36.4)	35 (63.6)	55	0.00 (0.966)	1.01 (0.64-1.59)
No	27 (36.0)	48 (64.0)	75		1.0
Depression					
Moderate-severe/severe	10 (23.8)	32 (76.2)	42	3.70 (0.054)	0.60 (0.34-1.06)
Subclinical/absent	91 (39.4)	140 (60.6)	231		1.0
Pollutant exposure (hours)					
More than 10	19 (42.2)	26 (57.8)	45	3.02 (0.221)	2.10 (0.82-5.40)
6-10	78 (37.5)	130 (62.5)	208		1.87 (0.77-4.58)
Less than 6	4 (20.0)	16 (80.0)	20		1.0
Air pollution diseases					
Yes	61 (39.6)	93 (60.4)	154	1.04 (0.310)	1.18 (0.86-1.62)
No	40 (33.6)	79 (66.4)	119		1.0
Noise pollution diseases					
Yes	47 (43.5)	61 (56.5)	108	3.26 (0.071)	1.33 (0.98-1.81)
No	54 (32.7)	111 (67.3)	165		1.0
Headache					
Yes	31 (45.6)	37 (54.4)	68	0.32 (0.571)	1.14 (0.72-1.81)
No	16 (40.0)	24 (60.0)	40		
Hearing Loss					
Yes	8 (27.6)	21 (72.4)	29	4.09 (0.043)	0.56 (0.30-1.05)
No	39 (49.4)	40 (50.6)	79		

PR = prevalence ratio.

The overall prevalence of pregnancy complications (moderate-severe) reported by these workers was 36.9% (Tables 1, 2, and 3).

### PREGNANCY COMPLICATIONS ASSOCIATED WITH SOCIODEMOGRAPHIC DATA AND HOUSEHOLD RESPONSIBILITIES

Statistically significant associations were identified (p < 0.05), showing a higher prevalence of pregnancy complications among those living in housing conditions other than a house, apartment, room, or boarding house (PR = 2.45, 95% CI 1.61-3.73) and among those responsible for the care of children or older people (PR = 1.10, 95% CI 1.01-1.20). Conversely, workers aged 60 years or older reported significantly fewer pregnancy complications (PR = 0.50, 95% CI 0.27-0.90) (Table 1).

A higher prevalence of pregnancy complications was also observed among workers who did not have a partner, those who had lower monthly incomes, those responsible for individuals requiring special care, those with more than 2 dependents, those who almost never or never felt unconcerned about leaving their children at home unsupervised, and those living in boarding houses or other types of housing (Table 1). Although they were not statistically significant associations, a lower prevalence of pregnancy complications was observed in workers older than 30 years and in those who belonged to blended, lone-parent, and single-parent families (Table 1).

### PREGNANCY COMPLICATIONS ASSOCIATED WITH OCCUPATIONAL, ENVIRONMENTAL, AND SUPPORT OR SOLIDARITY EXPOSURES

A statistically significant association was identified (p < 0.05), showing a 50% higher prevalence of pregnancy complications among those who had worked during their pregnancy (PR = 1.5, 95% CI 1.08-2.09) and among those who perceived discrimination by their colleagues (PR = 1.42, 95% CI 1.04-1.94) (Table 2).

Although there was no statistical significance, a higher prevalence of pregnancy complications was also identified in those with more than 20 years of seniority as a “ventera,” those who felt they had to work harder than their male peers, workers who did not perceive respect from their peers, and those who perceived discrimination by the state. These complications were less prevalent among those who worked 5 or fewer days a week (PR = 0.57) (Table 2).

### PREGNANCY COMPLICATIONS ASSOCIATED WITH WORKERS’ HEALTH CONDITIONS

#### Chronic degenerative diseases

Statistically significant associations were identified (p = 0.05), where the prevalence of pregnancy complications was lower in women who, at the time of the survey, had moderate-to-severe or severe depressive symptoms (PR = 0.60, 95% CI 0.34-1.06) and in those who reported hearing loss (PR = 0.56, 95% CI 0.30-1.05) (Table 3).

Although the associations were not significant, a lower prevalence of pregnancy complications was observed among workers with overweight/obesity and those who reported having a diagnosis of diabetes at the time of data collection for this study (Table 3).

#### Diseases or symptoms related to environmental exposures and conditions

Although the associations were not significant, a higher prevalence of pregnancy complications was identified among workers who reported being exposed to environmental pollution for more than 10 hours or between 6 and 10 hours daily, among those who reported having illnesses related to air pollution, and among those who had become ill due to environmental noise. This increased prevalence was particularly marked in workers who reported having headaches frequently (PR = 1.14) (Table 3).

### SOCIODEMOGRAPHIC DATA AND HOUSEHOLD RESPONSIBILITIES THAT CONTRIBUTE TO THE EXPLANATION OF PREGNANCY COMPLICATIONS

When sociodemographic data and household responsibilities with p-values < 0.25 were simultaneously included to assess their collective contribution to explaining the prevalence of pregnancy complications among these workers, only two of these factors remained statistically significant (p < 0.05) and explanatory. However, directionality in the analysis for those living in a room/boarding house changed from being a factor associated with fewer pregnancy complications in bivariate analysis (_Crude_PR [cPR] = 0.65, 95% CI 0.44-0.97) to explaining a higher prevalence of complications (_Adjusted_PR [_A_PR] = 3.78, 95% CI 1.20-11.91). Older age was also a factor associated with a lower prevalence of complications during pregnancy. In this regard, those aged 60 years or older had a lower prevalence of complications (p = 0.042) than those aged 18 to 29 years (_c_PR = 0.5, 95% CI 0.27-0.9; _A_PR = 0.08, 95% CI 0.008-0.912) (Table 4).

**Table 4:** Sociodemographic data and household responsibilities that contribute to the explanation of pregnancy complications in informal workers called “venteras,” Medellín, 2016 (n = 291)

Characteristics	CrudePR	95% CI		p-value	Adjust.PR	95% CI	
		LL	UP			LL	UL
Age - 18 to 29 years (Rc)							
30-44	0.66	0.40	1.08	0.200	0.48	0.15	1.48
45-59	0.69	0.43	1.11	0.449	0.64	0.20	2.06
≥ 60	0.50	0.27	0.90	0.042	0.08	0.00	0.91
Financially dependent persons - ≤ 2 (Rc)							
> 2	1.26	0.92	1.70	0.362	1.39	0.69	2.81
Type of family - Nuclear (Rc)							
Extended	0.97	0.62	1.52	0.502	0.69	0.24	1.99
Reconstituted	0.90	0.59	1.37	0.746	1.18	0.44	3.13
Lone parent	0.50	0.25	1.01	0.212	0.22	0.02	2.40
Single parent	0.71	0.46	1.09	0.274	0.59	0.23	1.52
Blended	0.32	0.05	1.99	0.648	0.56	0.05	6.77
Housing type - House, apartment (Rc)							
Room-boarding house-other	0.65	0.44	0.97	0.023	3.78	1.20	11.91
Responsible for children or older people - No (Rc)							
Yes	1.1	1.01	1.20	0.314	0.41	0.07	2.35
In charge of people who require special care - No (Rc)							
Yes	1.33	0.92	1.91	0.749	1.15	0.50	2.62
Receives state subsidy - No (Rc)							
Yes	1.32	0.91	1.92	0.358	1.49	0.64	3.47

Rc = reference category; LL = lower limit; UL = upper limit; PR = prevalence ratio.

Finally, it is important to note that, although some of the conditions explored here did not significantly contribute to the explanation, they did have an impact on the prevalence of pregnancy complications. A higher prevalence of complications was observed among women living in reconstituted families and those who reported receiving state subsidies (Table 4).

### WORKING CONDITIONS, SUPPORT AND SOLIDARITY THAT CONTRIBUTE TO THE EXPLANATION OF THE HIGHER PREVALENCE OF PREGNANCY COMPLICATIONS

Only 1 out of the 7 variables evaluated showed explanatory capacity (p < 0.05): women who reported having worked during pregnancy experienced 80% more pregnancy complications. This condition retained its directionality and increased its magnitude and strength of association (_c_PR = 1.50, 95% CI 1.08-2.09; _A_PR = 1.80, 95% CI 1.07-3.03) (Table 5).

**Table 5:** Working conditions, support and solidarity at work that contribute to the explanation of pregnancy complications in informal workers called “venteras,” Medellín, 2016 (n = 291)

Characteristics	CrudePR	95% CI		p-value	Adjust.PR	95% CI	
		LL	UL			LL	UL
Worked during pregnancy - No (Rc)							
Yes	1.50	1.08	2.09	0.027	1.80	1.07	3.03
Working days per week - 5 days or less (Rc)							
6 or 7	0.57	0.33	1.01	0.117	0.25	0.04	1.41
Feels she has to work harder than her male peers - No (Rc)							
Yes	1.23	0.90	1.68	0.620	1.14	0.68	1.92
Perception of respect from peers - Yes (Rc)							
No	1.30	0.88	1.94	0.341	1.53	0.64	3.67
Perception of peer support - Yes (Rc)							
No	0.97	0.68	1.40	0.350	0.72	0.36	1.43
Perception of peer discrimination - No (Rc)							
Yes	1.42	1.04	1.94	0.139	1.60	0.86	2.97
Perception of discrimination by the state - No (Rc)							
Yes	1.28	0.92	1.77	0.342	1.18	0.76	2.21

Rc = reference category; LL = lower limit; UL = upper limit; PR = prevalence ratio.

Although not statistically significant, the multivariate model indicated that women who felt they had to work harder than their male peers experienced more pregnancy complications than those without such perception. In addition, complications were more prevalent among workers who perceived disrespect, felt discriminated against by peers, and experienced discrimination by the state (Table 5).

### HEALTH CONDITIONS THAT CONTRIBUTE TO THE EXPLANATION OF PREGNANCY COMPLICATIONS

Finally, an increase in the prevalence of pregnancy complications (p < 0.05) was observed in relation to moderate/severe depressive symptoms (_A_PR = 15.02, 95% CI 1.34-167.79), as well as among workers who had been exposed to environmental pollutants at their workplace for more than 10 hours (_A_PR = 8.11, 95% CI 8.04-214.09). The latter condition regained its explanatory capacity after adjusting for body mass index, diabetes, hypertension, and hearing loss (Table 6).

**Table 6:** Health conditions that contribute to the explanation of pregnancy complications in informal workers called “venteras,” Medellín, 2016 (n = 291)

Conditions	CrudePR	95% CI		p-value	Adjust.PR	95% CI	
		LL	UL			LL	UL
BMI - Low-normal weight (Rc)							
Overweight/obesity	0.77	0.56	1.05	0.656	1.24	0.35	4.32
Diabetes - No (Rc)							
Yes	0.54	0.22	1.34	0.238	2.08	0.52	8.33
Depression - Subclinical/absent (Rc)							
Severe-moderate	0.60	0.34	1.06	0.028	15.02	1.34	167.79
Pollutant exposure - Less than 6 hours (Rc)							
Between 6 and 10	1.87	0.77	4.58	0.139	0.65	0.18	2.27
More than 10	2.10	0.82	5.40	0.213	8.11	8.04	214.09
Hearing Loss - No (Rc)							
Yes	0.56	0.30	1.05	0.114	2.85	0.78	10.42

Rc = reference category; BMI = body mass index; LL = lower limit; UL = upper limit; PR = prevalence ratio.

Although the other characteristics or conditions included in the model did not significantly contribute to explaining higher pregnancy complications for this group of women, complications were more frequent among those who had overweight/obesity, diabetes, and hearing loss (Table 6).

## DISCUSSION

While this study represents a valuable contribution to the recognition and advancement of the understanding of pregnancy complications experienced by female workers, whose experiences may be similar to those of other informal female street vendors across the continent, it is important to note that the prevalence of these complications was self-reported by each worker. Furthermore, they were not specifically asked about their age at the time of experiencing such complications, as the event (pregnancy complications) had already occurred for all of them. These study limitations and issues should be further explored in future studies and analyses.

Complications during pregnancy were significantly less prevalent in women aged 60 years or older. Regarding the Millennium Development Goal related to maternal mortality, Colombia has made significant progress that can be explained, among other factors, by a reduction in pregnancy rates, expanded access to institutional childbirth care by qualified personnel, the development of technical standards and care guidelines for early detection of pregnancy complications, and standardized management for vaginal delivery and labor dystocia.^13^ Additionally, there has been an increased availability of consultations and better access to care for women during pregnancy and postpartum.

Furthermore, the age at the time of pregnancy is a variable to be considered, as age extremes are a risk factor for complications. Pregnancy in women over the age of 40 poses an increased risk of preterm delivery, preeclampsia, gestational diabetes, cesarean section, and intrauterine death.^14^ Similarly, pregnancy in those under 18 years of age has a higher frequency of anemia, preterm delivery, and cesarean section,^15^ a situation that should be considered relevant since, in previous decades, women were likely to become pregnant at a younger age.

The literature reports the responsibility of caring for children under 5 years of age or individuals with disabilities as a psychosocial risk factor.^16^ Although this was not statistically significant in the present study, it is noteworthy, from a public health perspective, that nearly 50% of the workers had 2 or more dependents and also experienced a higher prevalence of pregnancy complications. A significantly higher prevalence of complications (p < 0.05) was observed among those responsible for the care of children, older adults, or individuals requiring special care.

Studies have reported that women from nuclear families are at increased obstetric risk, followed by those from nuclear families in the phase of modern dispersion and from extended families in the dispersion phase. In addition, this risk tends to decrease in nuclear families with a traditional marital status.^17,18^ These findings differ from the results of the present study, where women from reconstituted families had a higher prevalence of pregnancy complications. The lack of stable housing or a homeless condition are associated with a high risk of complications for both the mother and the child,^19^ and Spanish guidelines for pregnant women in confinement situations emphasize maintaining good ventilation and cleaning contact surfaces.^20^ Although there are no data determining the risk of obstetric complications based on the type of housing, this study contributed to explaining the higher prevalence of pregnancy complications in women living in rooms/shared housing or boarding houses.

Conversely, living in low socioeconomic status housing during pregnancy is associated with poor prenatal care, which increases the risk of adverse outcomes such as obstetric hemorrhage, miscarriage, preterm labor, preeclampsia, eclampsia, cesarean section, and gestational diabetes.^21,22^ In this study, 75.6% of the women lived in low and very low socioeconomic status housing, a condition that contributed to explaining the high prevalence of complications during pregnancy.

Regarding work during pregnancy, despite some recommendations aimed at preventing complications,^23^ there are no recommendations specific to women engaged in subsistence work. Working hours and shifts should be tailored to each woman, with adjustments to reduce hours, allowing for prenatal visits and avoiding night shifts or long working hours, especially if the woman feels incapable due to physical or mental health issues.^23^

No specific recommendations were identified in the literature regarding work for pregnant women in the informal sector; however, general guidelines have been proposed for any type of employment. Despite this, certain working conditions that involve risks during pregnancy cannot necessarily be avoided. In the case of informal workers such as the “venteras,” many women continue working for economic reasons, openly stating that they must work during the day to be able to eat at night.

It has been shown that discrimination against pregnant women in the workplace is highly prevalent worldwide. However, its effects on the health of mothers and their children are inconclusive.^23^ Discrimination as perceived by mothers has been indirectly linked to an increase in postpartum depressive symptoms, LBW, lower gestational age, and higher number of newborn medical visits.^24^ In this study, workers who felt they had to work harder than men, who perceive disrespect from their colleagues, who felt discriminated against by their peers, and who experienced greater discrimination by state authorities also had a higher prevalence of pregnancy complications.

Overweight and obesity are conditions associated with complications such as gestational diabetes, preeclampsia, preterm labor, assisted delivery, cesarean section, infections, and postpartum hemorrhage.^25^ For the workers participating in this study, being healthy during pregnancy is a difficult premise to meet, given the high prevalence of overweight/obesity in this population. Although overweight/obesity in informal workers has been linked to working up to 10 hours a day, 6 days a week, and having greater seniority in their profession, the relationship between this condition and pregnancy complications had not yet been explored. In this study, after adjusting for the variables analyzed in the multivariate models, overweight and obesity appeared as factors contributing to a higher prevalence of pregnancy complications, although this association was not statistically significant, similar to the case of diabetes mellitus.

Diabetes mellitus is a condition that needs to be identified and managed appropriately, especially in women who develop it during pregnancy.^26^ This disease can trigger complications such as preeclampsia, maternal and neonatal infections, postpartum hemorrhage, cesarean section, polyhydramnios, hyperglycemic coma, stroke, and ketoacidosis, among others.^26^ Hypertensive disorders during pregnancy have been identified as common complications and considered a major cause of maternal and/or fetal morbidity and mortality,^27^ leading to conditions such as pulmonary edema, heart failure, placental abruption, disseminated intravascular coagulation (DIC), and HELLP (hemolysis, elevated liver enzymes, low platelet count) syndrome. Specifically, hypertension complicates approximately 10% of pregnancies.^28^

Depression is the most common psychiatric morbidity during pregnancy. In Colombia, according to a study conducted by Martínez-Paredes & Jácome Pérez,^29^ the prevalence of depression during pregnancy is approximately 19%. However, a study conducted by Bonilla-Sepúlveda^30^ involving adolescent mothers in a Colombian city showed a higher prevalence of depression, reaching up to 32.8%. Complications associated with this condition include preeclampsia, preterm birth, fetal neurodevelopmental impairment, low Apgar score, LBW for gestational age, and an increased risk of the consumption of substances harmful to the fetus. In the present study, after adjusting for depressive symptoms along with other variables, depression was found to be a condition that significantly contributed to the higher prevalence of pregnancy complications in our participants.

In this study, more than 80% of the workers with moderate/severe depressive symptoms had an informal street vending or semi-stationary vending shop. Additionally, 95% of them worked 6 to 7 days a week, and 3 out of 4 workers with pregnancy complications worked more than 8 hours a day. On the other hand, working in a noisy environment during pregnancy can affect maternal hearing and increase stress levels, leading to changes in fetal development. Therefore, it is important to emphasize that pregnant women should not be routinely exposed to noise levels higher than 115 dBA, even if they are using hearing protection.

Repetitive and loud noises should be avoided to prevent disturbances in fetal development.^6,23^ However, this was challenging for the workers participating in this study, who, due to their working conditions, reported having to shout to communicate and carry out their tasks on the streets and sidewalks of the city. In these areas, air and noise pollution are high due to heavy vehicle traffic, which is common not only in the city of Medellín but also in other cities across the country and continent.

## CONCLUSIONS

While it is true that some actions could be implemented to help these workers avoid pregnancy complications, such as promoting prenatal care and encouraging healthy lifestyle habits, it is equally important to consider their living and working conditions when implementing these practices. Achieving this goal requires comprehensive and coordinated actions from the state, the working population, and society, ensuring that these actions align with the lifestyles and working conditions of women engaged in subsistence street vending in urban areas.
